# *Bacillus* Cellulase Molecular Cloning, Expression, and Surface Display on the Outer Membrane of *Escherichia coli*

**DOI:** 10.3390/molecules23020503

**Published:** 2018-02-24

**Authors:** Daehwan Kim, Seockmo Ku

**Affiliations:** 1 Laboratory of Renewable Resources Engineering, Purdue University, West Lafayette, IN 47907, USA; kim1535@purdue.edu; 2 Department of Agricultural and Biological Engineering, Purdue University, West Lafayette, IN 47907, USA; 3 Fermentation Science Program, School of Agribusiness and Agriscience, College of Basic and Applied Sciences, Middle Tennessee State University, Murfreesboro, TN 37132, USA

**Keywords:** *Bacillus licheniformis*, cellulase, ice nucleation protein, *Pseudomonas syringae*, surface anchoring, whole cell catalysis

## Abstract

One of the main challenges of using recombinant enzymes is that they are derived from genetically-modified microorganisms commonly located in the intracellular region. The use of these recombinant enzymes for commercial purposes requires the additional processes of cell disruption and purification, which may result in enzyme loss, denaturation, and increased total production cost. In this study, the cellulase gene of *Bacillus licheniformis* ATCC 14580 was cloned, over-expressed, and surface displayed in recombinant *Escherichia coli* using an ice-nucleation protein (INP). INP, an outer membrane-bound protein from *Pseudomonas syringae*, was utilized as an anchor linker, which was cloned with a foreign cellulase gene into the pET21a vector to develop a surface display system on the outer membrane of *E. coli*. The resulting strain successfully revealed cellulase on the host cell surface. The over-expressed INP-cellulase fusion protein was confirmed via staining assay for determining the extracellular cellulase and Western blotting method for the molecular weight (MW) of cellulase, which was estimated to be around 61.7 kDa. Cell fractionation and localization tests demonstrated that the INP-cellulase fusion protein was mostly present in the supernatant (47.5%) and outer membrane (19.4%), while the wild-type strain intracellularly retained enzymes within cytosol (>61%), indicating that the INP gene directed the cellulase expression on the bacteria cell surface. Further studies of the optimal enzyme activity were observed at 60 °C and pH 7.0, and at least 75% of maximal enzyme activity was preserved at 70 °C.

## 1. Introduction

In previous decades, biotechnological innovations have made it possible to utilize a variety of natural resources as raw materials in the manufacturing of commercial products [1]. Due to the development of technology for commercial purposes and the needs of consumers, the spectrum of uses for various raw materials has grown exponentially [2]. Among these natural sources, molecules derived from plant resources have become an important aspect of creating value-added products in the global market [3]. One of the molecules that is essential for plant processing, especially upstream and/or downstream processing, is glycosilase, due to its applications over a broad range of substrates, specific catalysts, low energy consumption, limited toxic molecule generation, and high-end product yield [4,5,6,7,8]. Of the various glycosilases, one of the most widely used enzymes for bioprocessing for plant resources is cellulase.

Cellulase, a mixture of endo-glucanase, exo-glucanase, and β-glucosidase, is capable of cleaving the β-1, 4-d-glucan bond in cellulose matrices to liberate monomeric sugars, such as glucose and some cellobiose. This glycosilase has been widely utilized in industrial applications and diverse commodities, including second-generation biofuels, foods, and biochemical resources [9,10,11]. Despite various advantages, cellulase has several limitations: (i) its bio-catalytic process should be conducted under delicate experimental conditions (e.g., pH and temperature); (ii) cellulase is easily inhibited by plant derived-inhibitory compounds generated from substrates and/or physical shear stress in a reactor; and (iii) cellulase is costly, and impacts the total cost of bioprocessing. 

To increase the production of cellulase and minimize the total cost of cellulose industrial applications, recombinant microbial cellulase production via genetic engineering was proposed and rapidly developed [4]. For example, *Trichoderma* and *Aspergillus* microorganism species are widely used in commercial cellulases [12] due to their effective and varied endoglucanase production [13,14]. These strains are strongly preferred for genetic engineering cellulase production, which requires additional steps to recover and purify the target enzymes due to the limitations of the cell’s intercellular microbial enzymes [8]. Moreover, cell recovery and purification processes may contribute to decreased enzyme activity and increased production cost. In order to overcome these issues, the bacterial cell surface display of heterologous proteins on the outer membrane of Gram-negative bacteria [15], Gram-positive bacteria [16], and archaebacteria [17] has also been investigated with anchoring motif proteins. Surface display systems are generally constructed by encoding targeted gene and anchoring sequences. Anchoring sequences are provided by outer membrane proteins and/or surface structural proteins, such as ice nucleation protein [15,18,19,20,21], frimbriae [22,23], β-auto transporters [24], lipoproteins [25], and secretory proteins [20,21,26]. Surface-displayed functional enzymes on the outer membrane of the microorganism are essential, low-cost biocatalysts that could be utilized in the development of various applications, including whole cell systems [27,28,29,30], environmental bio-adsorbents [31,32], live vaccines [33,34,35], and immunological antibodies [36,37,38,39]. The use of successfully-expressed, surface-displayed fusion proteins led to efficiently and economically eliminating a step in enzyme purification, and the cells with extracellular enzymes had less mass transfer resistance when the enzymes exhibited toward the corresponding substrates and products allowing better product recoveries and downstream processing [40,41].

The ice nucleation protein (INP), an outer membrane-bound protein from *Pseudomonas syringae*, formed ice crystallization in super-cooled water [42,43]. INP is considered a promising anchoring protein for cell surface display because not only it is stably expressed during cell culture, but it also features internal repeating domains whose length can be easily manipulated [21,44,45,46]. INP is composed of three structural N-terminal domains, thought to anchor foreign protein to the cell surface. The C-terminal domains have relatively hydrophilic properties, and the central domains consist of repeated amino acid sequences responsible for ice crystal formation [15,47].

In this study, the gene cloning and over-expression of *Bacillus* cellulase on the outer membrane of *E. coli* were investigated. We used the INP of *P. syringae* KCTC 1832 as an anchor protein and cloned the cellulase gene of *Bacillus licheniformis*. Since the targeted cellulase protein has a high molecular weight, most of the enzyme was retained within the cytosol region. The surface-expressed cellulase activity was confirmed via Congo red staining on carboxylmethylcellulose (CMC)-containing medium, and the molecular weight of the recombinant cellulase was identified using the Western blotting method. Furthermore, we studied the effects of temperature and pH on purified cellulase activity, and also evaluated the optimal conditions for recombinant cellulase.

## 2. Results and Discussion

### 2.1. Cloning and Expression of the INP-Cellulase Gene

**Figure 1:**
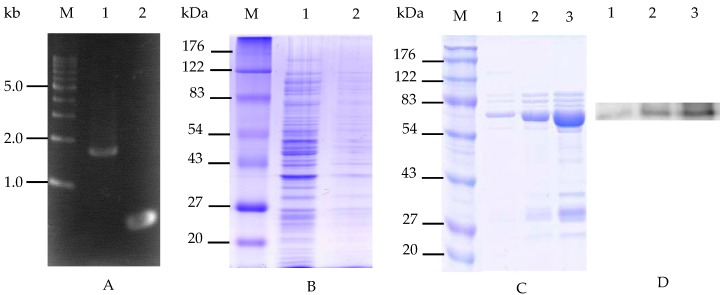
Targeted gene insertion and expression in *E. coli*. (**A**) The gel electrophoresis of amplified PCR cellulose products from *B. licheniformis* ATCC 14580 (lane 1) and INP from *P. syringae* KCTC 1832 (lane 2). M: 1 kb DNA marker; (**B**) SDS-PAGE analysis of the recombinant cells; M: standard protein size marker (molecular biomasses in kilodaltons), lane 1: the supernatant fraction of recombinant cell culture medium, lane 2: the total cell lysates of recombinant cell; (**C**) The purified fusion proteins following Ni-nitrilotriacetic acid (NTA)-sepharose resin treatment; M: standard protein size marker (kDa), lane 1: imidazole concentration of 20 mM in the binding buffer, lane 2: imidazole concentration of 50 mM in the binding buffer, lane 3: imidazole concentration of 100 mM in the binding buffer; (**D**) Western blot analysis of the purified fusion protein from SDS-PAGE results probed with anti-His-tag antibody, respectively.

To explore the anchoring cellulase on the outer membrane of *E. coli*, the stop codon (TGA) in the INP gene of *P. syringae* KCTC 1832 was deleted and digested by Nde I and EcoR I restriction enzymes in order to clone the INP gene into the pET21a vector and construct the *Bacillus* cellulase gene. The resulting INP gene was initially cloned into the N-terminal His tag region of the pET21a vector, which contained the T7 promoter (pET-INP) [20]. The cellulase, which consisted of 1683 nucleotides that encode 560 amino acids with an expected molecular weight of 61.7 kDa, was isolated from *B. licheniformis* ATCC 14580 and fused to the C-terminal of INP in the vector, which was cloned in the previous step. The recombinant genes were constructed in the following order: T7 promoter, INP gene, and *Bacillus* cellulase gene. Verification of the INP and cellulase genes was performed with PCR amplification. The amplified bands of cellulase and INP were located near 1.6 kb and 0.5 kb, respectively (Figure 1A). The insertion of the cellulase gene fragment was carried out on the multiple cloning site of pET-INP with restriction sites Nde I and Not I (pET-INP-cellulase). The inserted INP and cellulase genes were confirmed via sequence analysis of the original and transformed strands. The strands’ INP and cellulase gene sequence identities were 100% and 98% conserved, respectively (unpublished observations). After the expression of the recombinant cells with 2 mM IPTG, the expression of INP-cellulase fusion protein was identified by SDS-PAGE (Figure 1B). Multiple bands were identified within the culture supernatant and total crude cell extract (Figure 1B, lanes 2 and 3), which confirmed that most of the recombinant enzyme proteins were located in the extracellular supernatant, rather than in the cell. The expected molecular weight of the cellulase gene was 61.7 kDa, plus the INP fusion protein (19.7 kDa) and poly-histidine tag (1 kDa) [20,48,49]. The expressed fusion protein presented thick mobility bands corresponding to around 80 kDa (Figure 1C). The purified fusion protein was clearly detected using Ni-NTA chromatography, which indicated that the cellulase gene was successfully constructed in the C-terminal (His)_6_-tag regions. When the imidazole concentrations were increased from 20 mM to 100 mM for the further purification of the His-tagged proteins, the targeted proteins were eluted (Figure 1C, lanes 1–3). Further observation via Western blotting also confirmed that the recombinant fusion protein was properly purified and observed with the anti-His-tag antibody (Figure 1D).

**Figure 2:**
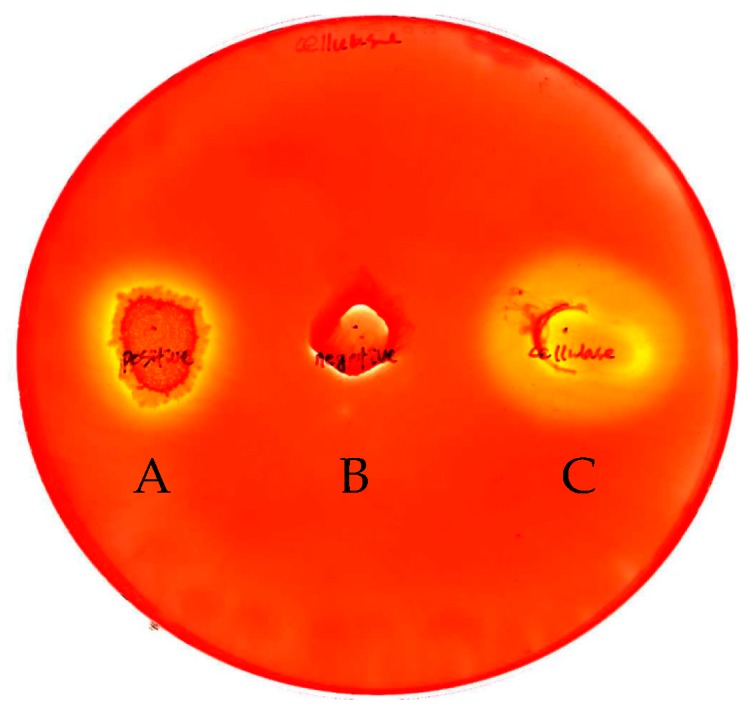
Congo-red staining assay of wild-type *B. licheniformis* (**A**); non-cellulase producing *E. coli* (**B**); and recombinant *E. coli* harboring INP-cellulase genes (**C**); all of which were topped and cultivated on agar medium of 0.5% CMC at 30 °C for 18 h. The hollow clear zones around the colonies indicate the degradation of CMC as a result of the cellulolytic enzymes.

To verify the expression of the INP-cellulase protein on the *E. coli* surface membrane, CMC (carboxylmethylcellulose) degradations with clear zone formations around the colonies were detected using Congo-red staining. Negative and positive controls of non-cellulase producing *E. coli* and wild-type *B. licheniformis* strains were used, and compared to the recombinant cell results (Figure 2). The hollow zone diameters of wild type *B. licheniformis*, *E. coli*, and INP-cellulase recombinant cells were 0.5 mm, 0 mm, and 22 mm, respectively. These results highlighted that the wild-type strain partially released cellulase extracellularly, while the INP-cellulase fusion proteins were functionally expressed and transported to the outer cell membranes by hydrolyzing more CMC than the wild-type strain (Figure 2A,C). The control test with *E. coli* did not show a clear zone around the colony. 

To further characterize the cellulase activity of recombinant cells harboring INP-cellulase, the cells were fractionated into the supernatant, membrane, periplasmic, and intracellular fractions. The expression and localization of the fusion protein was necessary to prove that the INP protein was able to translocate cellulase across the cell membrane and exhibit itself on the host cell surface as an anchor linker. The fractionated solution (including the cellulase activity) was determined using 0.5% (*w*/*v*) CMC as a substrate (Table 1). The wild-type enzyme accounted for around 84% of the cellulase activity and was retained intracellularly in the periplasm (22.7%) and cytoplasm (61.3%, intracellular), whereas most of the cellulase activity of the recombinant cells was detected extracellularly in the culture medium (47.5%), periplasmic (31.2%), and membrane (19.4%) fractions, respectively (Table 1). These results suggest that the fusion protein was successfully translocated to the outer cell membranes. *B. licheniformis* is a Gram-positive bacteria, which is partially able to produce extracellular enzymes [50]. In the current work, some of the wild-type cellulase was identified in the culture medium (17.2% in supernatant) and membrane (1.4%) fractions; however, these components were 18–19% portions of the total cellulase activity, which concurs with the previous study [50]. Other work on cloning foreign genes and fusion protein expression reported that enzymes were commonly localized in the cytoplasm of the host cells [51,52,53], which also confirms the INP’s capability to translocate the foreign proteins to the outer membranes of the host cells.

### 2.2. Characterization of Recombinant Cellulase

**Figure 3:**
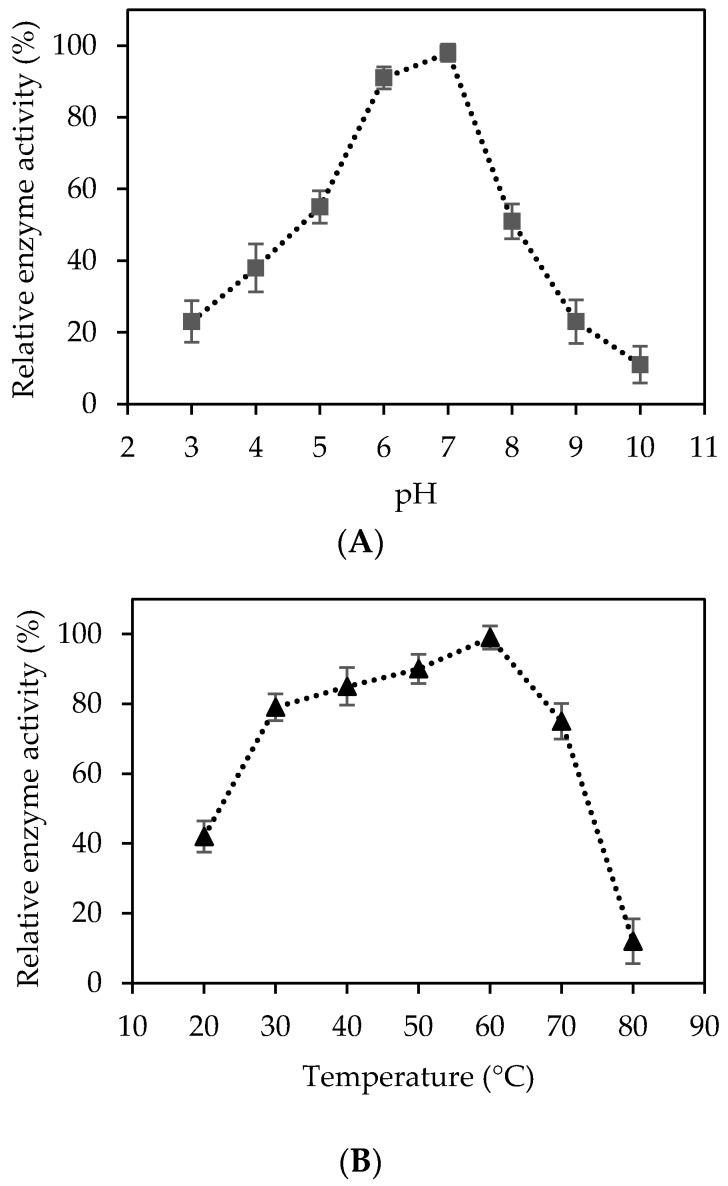
Relative cellulase activity analysis of various pH and temperature conditions. (**A**) Enzymatic hydrolysis of 0.5% CMC was carried out at 50 °C for 3 h with an agitation of 200 rpm; (**B**) Enzymatic hydrolysis of 0.5% CMC was carried out at pH 7.0 for 3 h with an agitation of 200 rpm. Error bars indicate the standard deviation of triplicated tests.

In order to determine the optimal cellulolytic temperature and pH, the enzymatic hydrolysis of CMC (0.5% *w*/*v*) was performed at temperatures of 20–80 °C and 3.0–10.0 pH. The optimal conditions of purified cellulase was identified as 50 °C and pH 7.0. Enzyme activity was relatively higher (>90%) at 50 °C–60 °C and pH values of 6.0–7.0 than the results of other experimental conditions (Figure 3A,B). One interesting observation was that the cellulase activity was still sustained at around 75% at 70 °C, which suggested that *Bacillus* cellulase was thermostable (Figure 3B). Similar studies with recombinant phytase from the same *Bacillus licheniformis* species have demonstrated that the recombinant phytase not only demonstrated an optimal enzyme activity at 75 °C, but was also stable up to 90 °C while maintaining approximately 40% of its enzyme activity [54]. While recombinant phytase was remarkably sensitive to pH below 5.5 and above 8.0, it displayed metal-resistant properties, especially to the calcium ion [54]. Bischoff et al. [55] reported that purified endoglucanase from the *B. licheniformis* B-41361 strain had an optimal enzyme activity at 65 °C and was mostly stable at 60 °C with more than 90% of its enzyme activity, which indicated the moderate thermo-stability of the enzyme. The biochemical properties of the recombinant cellulase in this work were consistent with previous research studies on the *B. licheniformis* strain genes’ ability to tolerate a high temperature range of 50–70 °C. This tolerance is notably advantageous for further research studies on other strain genes and potential industrial applications. Many attempts have found that an effective cellulosic biomass conversion requires robust and stable cellulolytic enzymes at high temperatures and extreme pH conditions in order to improve the final yield, productivity, and the cost of industrial processes [56,57]. 

*B. licheniformis* is considered one of the attractive microorganisms because its biochemical and phenotypic properties are extensively close to the well-known microbial strain *Bacillus subtillis*. One of the main features of the *B. licheniformis* genome is that it does not have a replication terminator protein (rtp) with a deficient rtp function; however, it does include the encoding genes arginase, asparaginase, ariginine deiminase, and glutaminase. These host genes are capable of metabolizing imino and amino acids [50]. These strain properties have been widely studied and applied to various bio-chemical industrial fields as a detergent agent [52], α-amylase [58,59], peptide antibiotics [60,61], fungal pathogens inhibitors [62], β-mannanase [63,64], cycloglucosyltransferase [65], pectinase [66,67], pentosanase [68], and phytase [54,69,70]. Although several *B. licheniformis* studies have contributed to better understanding of the strain, the following aspects of the enzyme have yet to be examined: the cloning of *B. licheniformis* cellulase genes, its *E. coli* cell-surface display, and numerous enzymatic characteristics. The anchoring fusion protein system has promising applications as a whole cell biocatalyst for simultaneous saccharification and fermentation (SSF) [27,71,72] and a consolidated bioprocessing of enzyme production. Therefore, this study provides an extended understanding of the recombinant cellulolytic enzyme and its possible surface display system application in order to overcome the current limitations of purification, immobilization, and extraction.

## 3. Materials and Methods

### 3.1. Materials

The genomic DNA extraction bacteria kit, DNA purification kit, and plasmid mini-prep kit were purchased from Invitrogen (Grand Island, NY, USA). The Taq DNA polymerase, T4 DNA ligase, pET21a plasmid vector, PCR reagents, restriction enzymes, and DNA ladder marker were purchased from Takara Bio (Madison, WI, USA). *B. licheniformis* ATTC 14580 and the constructed INP gene without repeating regions (538 bp, 179 amino acid residues) were provided by the Korean Culture Center of Microorganisms (Seoul, Korea) and Korea Research Institute of Bioscience and Biotechnology (KRIBB, Daejeon, Korea), respectively. All other chemicals and reagents, including CMC and 3,5-dinitrosalicylic acid (DNS), were purchased from Sigma Aldrich (St. Louis, MO, USA). 

### 3.2. Bacterial Strains, Plasmids, and Culture Conditions

The *E. coli* BL21DE3 [B F^–^ *ompT gal dcm lon hsdS_B_*(*r_B_*^–^*m_B_*^–^) λ(DE3 [*lacI lacUV5*-*T7p07 ind1 sam7 nin5*]) [*malB*^+^]_K-12_(λ^S^)] (Invitogen, New York, NY, USA) strain was utilized as the host cell for the surface display expression of the fusion proteins. *B. licheniformis* ATTC 14580 was used as the cellulase gene host. For the chromosomal DNA isolation of *B. licheniformis*, 2 mL of stock solution was inoculated into 500 mL Erlenmeyer flasks in the presence of Luria-Bertani (LB) medium (1% tryptone, 0.5% yeast extract, 1% sodium chloride, pH 7.0) and incubated overnight at 37 °C and 200 rpm. To express the recombinant enzyme under the T7 promoter combined with the lac operon system, the cells were grown in the LB medium containing ampicillin (100 μg/mL) at 37 °C and 200 rpm [20,27]. Once the cell concentration of the culture medium reached OD 0.6–0.7 at 600 nm, the protein expression was induced at 25 °C by adding 0.5, 1, and 2 mM of isopropyl-β-d-thiogalactopyranoside (IPTG) for 18 h.

### 3.3. Plasmid Construction

To prepare the display of foreign fusion proteins, an INP gene from *P. syringae* KCTC 1832 was initially cloned into the pET 21a vector. The detailed preparation of the INP gene construction was described in previous works [20,73]. Briefly, to derive an anchoring fusion protein from the INP-foreign hybrid, the translational termination codon in the INP gene was deleted and digested by Nde I and EcoR I restriction enzymes, and then the digested INP fragment was inserted into the same pET 21a vector recognition sites. For further construction of the *B. licheniformis* encoding cellulase gene, the isolated cellulase gene was amplified via PCR and introduced into the sub-cloned pET-INP vector.

### 3.4. Cloning and Sequence Analysis

The chromosomal DNA-containing cellulase sequence of *B. licheniformis* ATCC 14580 was prepared and isolated with the JetFlex genomic DNA purification kit. The putative cellulase gene (1683 bp) from the genomic sequences was amplified via polymerase chain reaction (PCR) with two designed primers: forward primer 5′-GCTTGCGGCCGCTTGAGAGAAAAATGG-3′ and reverse primer 5′-GAGTGCGGCCGCAGGXGTGATTTTCACC-3′ (the recognition enzyme restriction site of Not I is underlined). Polymerase chain reactions were carried out in a Perkin-Elmer thermal cycler (GeneAmp PCR System 9700, Norwalk, CT, USA) for 30 cycles. Each cycle was composed of a denaturation stage at 94 °C for 45 s, an annealing stage at 56 °C for 2 min, and extension stage at 72 °C for 2 min. The amplified PCR fragments were purified via the PCR clean-up kit, digested by the Not I restriction enzyme, and then ligated into the corresponding restriction site of the digested vector. The ligation mixture was transformed into *E. coli* BL21DE3, and the transformants were screened on LB agar plates supplemented with ampicillin (100 μg/mL). The multiple nucleotide sequence analysis of the recombinant was confirmed with Clustal Omega software, and the amino acid sequence alignment was performed using BLASTP at the NCBI.

### 3.5. Cell Fractionation

To investigate the location of the expressed *E. coli* INP-cellulase fusion protein, cell fractionation steps for the intracellular and extracellular membranes of the enzymes were prepared according to previous studies [18]. Cell cultivation was performed overnight with IPTG, and the culture broth supernatant was centrifuged at 4 °C for 10 min and 6000 rpm. The collected cell pellets were washed twice with 0.9% sodium chloride solution and re-suspended in 0.05 M sodium phosphate buffer (pH 6.0) containing 25% sucrose. The mixture solution was centrifuged at 4 °C for 10 min and 6000 rpm. The harvested cells were re-suspended in 5 mL of distilled water and incubated at 4 °C for 10 min. The supernatant was recovered as the periplasmic fraction using a similar procedure. The cells were re-suspended in 0.05 M sodium phosphate buffer (pH 6.0) and disrupted with an ultrasonicator. The cell debris was separated via centrifugation at 4 °C for 10 min and 6000 rpm, and the resulting supernatant was followed by ultracentrifugation at 4 °C for 60 min and 15,000 rpm. The intracellular fraction was harvested from the resultant supernatant, and the membrane fraction of the remaining precipitate was re-suspended in the buffer.

### 3.6. Analytical Methods

A Congo-red staining assay was applied in order to measure the extracellularly-expressed cellulase from the surface-displayed fusion protein. Once the fresh colonies formed on the surface of the plate, the recombinant cells were incubated overnight on an LB agar (0.5% carboxylmethylcellulose (CMC)) plate at 37 °C. The overlaid agar medium was incubated at 37 °C for 6 h to allow for the cellulolytic digestion of CMC. The incubated medium was stained with Congo-red solution (0.2%, *w*/*v*) for 15 min and washed twice with 0.5 M sodium chloride solution. The generation of clear zones around the colonies was detected during the CMC hydrolysis.

The molecular weight and sensitivity of the INP-cellulase proteins were determined via sodiumdodecyl sulfate polyacrylamide gel electrophoresis (SDS-PAGE) as conducted in previous studies [18,74]. SDS-PAGE was performed using 10% polyacrylamide and then stained with Coomassi brilliant blue dye R-250. The (His) 6-tagged cellulase protein was bound to Ni-nitrilotriacetic acid (NTA)-sepharose resin (Qiagen, Hilden, Germany), pre-equilibrated with binding buffer, and washed with imidazole in a step-gradient manner range of 20 to 100 mM.

### 3.7. Enzyme Assay

The effects of pH and temperature on the recombinant cellulase activity were tested with 0.5% CMC from 30–70 °C and 5.0–8.0 pH. CMC (0.25 g/ 50 mL) was added to 0.05 M sodium citrate buffer containing 50 µL of the prepared cellulase, and cultivated at 50 °C for 72 h with an agitation of 200 rpm. The reducing sugars liberated from the cellulose were determined via colorimetric assay and the DNS method at 570 nm [75]. 

## 4. Conclusions

The *B. licheniformis* cellulase gene was fused to the N-terminus of the INP anchor, and the gene constructed as the *E. coli* cell surface display system. The fusion protein was successfully secreted extracellularly, and exhibited more than 98% of the cellulase activity. The results also confirmed a larger clear zone on CMC-containing agar medium than the wild-type strain results. The corresponding cellular fractionations and cellulase activity locations in wild-type and recombinant cells revealed that the cellulase was displayed on the cell membrane, and the INP anchoring system translocated the fusion protein across the membrane as an anchoring linker. The optimal conditions for the purified cellulase activity were 60 °C and pH 7.0; however, the cellulase sustained around 65% enzyme activity at 70 °C. Given that the INP-cellulase fusion protein was successfully expressed extracellularly, and its cellulase activity was stable at 60–70 °C, this cell surface display system may be a candidate for whole cell catalytic bioconversion and provide useful applications, such as cell immobilization and reusability.

## Figures and Tables

**Table 1:** Cell fractionation and location of cellulase activity in wild-type *B. licheniformis* and recombinant cell.

Cell Fractionation	Wild Type		Recombinant (INP-Cellulase)	
	(mU/mL)	(%) ^1^	mU/mL	(%) ^1^
Cell Culture Medium (Supernatant)	17.2	14.8	468.0	47.5
Periplasm	26.3	22.7	307.4	31.2
Cytoplasmic (Intracellular)	71.0	61.3	18.7	1.9
Membrane	1.4	1.2	191.1	19.4
Total Activity	115.9	100	985.2	100

^1^ (%) indicates the fractional cellulase activity per total cellulase activity.
